# Slower Decline in C-Reactive Protein after an Inflammatory Insult Is Associated with Longer Survival in Older Hospitalised Patients

**DOI:** 10.1371/journal.pone.0159412

**Published:** 2016-07-28

**Authors:** Maryam Barma, James A. Goodbrand, Peter T. Donnan, Mark M. McGilchrist, Helen Frost, Marion E. T. McMurdo, Miles D. Witham

**Affiliations:** 1 Ageing and Health, University of Dundee, Ninewells Hospital, Dundee, Scotland, United Kingdom; 2 Dundee Epidemiology and Biostatistics Unit, Dundee University Medical Research Institute, University of Dundee, Dundee, Scotland, United Kingdom; 3 Health Informatics Centre; Tayside Medical Science Centre, University of Dundee, Dundee, Scotland, United Kingdom; 4 Faculty of Health Sciences and Sport, University of Stirling, Stirling, Scotland, United Kingdom; University of Leicester, UNITED KINGDOM

## Abstract

**Background:**

Enhancing biological resilience may offer a novel way to prevent and ameliorate disease in older patients. We investigated whether changes in C-reactive protein (CRP), as a dynamic marker of the acute inflammatory response to diverse stressors, may provide a way to operationalize the concept of resilience in older adults. We tested this hypothesis by examining whether such changes could predict prognosis by identifying which individuals are at greater risk of 6-month mortality.

**Methods:**

Analysis of prospective, routinely collected datasets containing data on hospitalization, clinical chemistry and rehabilitation outcomes for rehabilitation inpatients between 1999 and 2011. Maximum CRP response during acute illness and CRP recovery indices (time and slope of CRP decay to half maximum, and to <50mg/L if peak values were greater than 50mg/L) was derived from biochemistry data. 6-month survival plots were conducted on quartiles of CRP recovery indices. Cox proportional hazards models were used to test univariate and multivariate predictors of 6-month mortality. Covariates included age, sex, number of medications, serum calcium, haemoglobin level, renal function, and the presence of previous myocardial infarction, stroke, chronic heart failure, COPD and diabetes.

**Results:**

3723 patients, mean age 84 years, were included. 1535 (41%) were male and 733 (20%) died during six-month follow-up. The lower an individual’s peak CRP reading, and the longer the time taken for their CRP to fall, the better their 6-month survival. The time for CRP to reach half of its maximum value was the best dynamic CRP index of survival (HR 0.93 per week, 95% CI 0.89 to 0.98; p = 0.004); this remained significant even after adjustment for maximum CRP level and covariates listed above.

**Conclusion:**

CRP recovery indices are associated with survival in older people; further work is required to explain differences in physiology between patients with a fast and slow CRP recovery.

## Introduction

Resilience has been described as the ability to withstand, adapt to, or recover from, a stressor. Although the concept of resilience is well established in engineering, psychology and ecology [1–2], less attention has been paid to the concept of biological resilience within medicine [3]. Although the concept of resilience may appear to have considerable overlap with the concept of frailty, they may not be entirely congruent. Measures of frailty are based in large part on static measures of function or deficit, whereas the concept of resilience embraces the dynamic response to a stressor. It may therefore be possible for a person to be frail yet resilient–or conversely to be non-frail yet lacking in resilience.

The study of resilience in ecological systems suggests four potential dimensions of resilience: latitude, resistance, precariousness and panarchy [2]. Of these dimensions, resistance would encapsulate the impact of a stressor, and the subsequent rate of recovery. Intercurrent illnesses are of particular concern in older people, frequently leading to permanent declines in function and often to a vicious cycle of further illness, hospitalisation, decline and death [4–5]. In order to make progress in studying the putative phenomenon of biological resilience, ways of operationalising and measuring the concept are required.

C-reactive protein (CRP) is a protein manufactured by the liver in response to high circulating IL-6 levels. Although it is targeted specifically at phosphocholine residues, it acts as a non-specific marker of systemic inflammation. CRP is an acute-phase protein, whose plasma concentration can rise by up to 1000 percent in inflammatory conditions [6]; raised levels are found in infection particularly when due to bacterial agents, malignancy, trauma and exercise. Levels rise within a few hours of infection and resolve with a mean half-life of nineteen hours [7]. It is already in use in routine clinical practice as a marker of infection and other stressors, as well as response to treatment. It thus provides an easily accessible exemplar measure to try and operationalise a measurement of resilience. Given that it reflects a person’s response to an infective or inflammatory stimulus, the rate of changes in CRP levels might therefore serve as a measure of the ‘resistance’ dimension of biological resilience. We therefore conducted an analysis of CRP changes using routinely collected data in a cohort of older people to test this hypothesis. We related the magnitude, and rate, of change in CRP levels to six-month mortality outcomes, using this as a clinical end-point for the ‘spiral of decline’ often seen in hospitalised, and presumably less-resilient, older adults.

Should our work show that indices of CRP response can indeed predict mortality, thus identifying the least resilient individuals, this investigation may establish the initial foundations for the development of a more rigorous resilience index. If resilience is indeed a concept distinct from frailty in older people, study of the phenomenon could provide new avenues for identifying those at risk of adverse outcomes, and more importantly, might suggest new ways to bolster resilience to protect older people against the adverse effects of intercurrent illness.

## Methods

### Study population

We analysed routinely collected, linked datasets on a cohort of older people who had undergone inpatient rehabilitation following acute illness over a 12 year period. The study population has been described before [8–9]. Individuals included in the study were all above 65 years of age, and had survived an initial acute hospital admission under the Medicine for the Elderly service in Ninewells Hospital, Dundee before undergoing inpatient rehabilitation under this service between 1999 and 2011. Linked datasets of routinely collected clinical data were held by the Health Informatics Centre, University of Dundee. The retrospective use of these data did not allow for individual consent to be obtained; management of these data is performed under generic ethics committee approval from the East of Scotland Research Ethics committee and the local Data Protection Officer. All patient information was de-identified and pseudoanonymised prior to analysis.

### Measures of CRP recovery

We hypothesised that the variables derived using maximum CRP response mounted, and subsequent serial measurements, would correspond to features of resilience. They may be able to offer insight as to the extent of deviation from the normal state of health and the ability to mount a response in the face of external stressors (such as infection or inflammation), whilst reflecting the rate of recovery for each individual. The variables that were derived for each patient were maximum CRP response, the time from maximum CRP reading to half-maximum, using linear interpolation of values from maximum to the first reading below half-maximum, the time from maximum CRP reading to 50mg/L, derived using a similar linear interpolation, the slope of decay to half-maximum, and the slope of decay to <50mg/L. This threshold was an arbitrary figure chosen to reflect current geriatric medicine practice as one likely to represent an acute stressor (values at or above this level are predictive of bacterial infection in older people) [10], and also one below which most practitioners would be happy that an episode of illness was improving. We included only those patients with a peak CRP reading during their acute admission, and at least one follow-up CRP reading. Not all patients had a follow up CRP reading below either half maximum or 50mg/L, hence numbers in analyses of these indices are lower than the total number of patients in the analysis. Similarly, not all patients had a maximum CRP reading of >50mg/L; those with lower maximum readings were not included in analyses of time or slope to 50mg/L.

### Covariates

Baseline demographic data included age, gender, hospitalisation dates and length of stay in rehabilitation. Date of death was obtained from the Scottish General Records Office, which maintains records from death certificates for all deaths in Scotland. Previous hospitalisation due to myocardial infarction, stroke, chronic heart failure or chronic obstructive pulmonary disease COPD was derived from Scottish Morbidity Record (SMR01) data on hospital discharges, using ICD-9 and ICD-10 codes. The presence of diabetes mellitus was derived from the Scottish Care Information–Diabetes Collaboration (SCI-DC) record, which contains details on all patients in Scotland diagnosed with diabetes mellitus. Estimated glomerular filtration rate (eGFR) was derived from the serum creatinine value after admission to rehabilitation, calculated using the MDRD4 equation [11]. Albumin and haemoglobin levels were obtained from linked, routinely collected laboratory data; the measurement after admission to rehabilitation was used. Linked dispensed community prescription data (held by HIC on all prescriptions dispensed outside hospital) was used to derive medication use. 20 point Barthel scores [12], a reliable and valid measure of disability, were held in the linked dataset; these were scored as part of routine care on admission to, and discharge from, inpatient rehabilitation.

### Statistical analyses

All analyses were conducted using SPSS v21 (IBM, New York, USA). Significance was defined as a 2-sided p<0.05. Descriptive statistics for population characteristics were derived as frequencies and proportions, mean values with standard deviations or as medians with inter-quartile ranges. Kaplan Meier survival analysis were used to determine the proportion of individuals alive at a given time during the six months following the date of peak CRP measurement during the acute admission to hospital. The cohort of patients was divided into quartiles for each derived measure of CRP, and the log-rank test was used to determine if the difference between groups was statistically significant. Cox proportional hazards models were used to test univariate and multivariate predictors of 6-month mortality. Hazard ratios were calculated to test the ability of each variable to predict death, whilst taking into account the effects of other independent variables. These included age, sex, number of medications, calcium, haemoglobin and albumin levels, eGFR as well as previous myocardial infarction, stroke, chronic heart failure, COPD and diabetes mellitus. Pearson’s correlation coefficients were calculated to determine the relationship between indices of CRP recovery and markers of rehabilitation, to test whether any effects on mortality might be mediated via effects on functional recovery.

## Results

3723 patients, mean age 84 years, were included. 1535 (41%) were male and 733 (20%) died during six-month follow-up. Baseline characteristics of the cohort are shown in Table 1. 3723 (85%) patients had a maximum CRP reading available during their acute admission, and 2496 (57%) individuals had a follow-up CRP reading of less than half of their maximum level, and were therefore included in the analyses for time and slope for CRP to fall to half of the individual’s maximum. We calculated the time and slope for CRP to fall to <50mg/L on 1858 (42.4%) people in whom maximum CRP exceeded 50mg/L.

**Table 1:** Baseline Population Characteristics of Cohort of Patients Analyzed (n = 3723 unless otherwise stated).

Mean age (years) (SD)	83.7 (7.5)
Male sex (%)	1535 (41.2)
Mean length of rehabilitation Stay (days) (SD)	52 (55)
Deaths by 6 months	733 (19.7)
Mean change in Barthel score during inpatient rehabilitation stay (SD)*	+3.9 (3.5)
Mean Barthel score at time of rehabilitation discharge (SD) (n = 1953)	14.0 (4.8)
Mean number of medications per patient (SD)	3.2 (3.5)
Mean serum calcium Level (mmol/L) (SD)	2.40 (0.13)
Mean eGFR (ml/min/1.73m^2^) (SD)	64 (27)
Mean haemoglobin (g/dL) (SD)	11.9 (1.9)
Previous myocardial infarction (%)	706 (19.0)
Previous stroke (%)	285 (7.7)
Chronic Heart Failure (%)	371 (10.0)
Chronic obstructive pulmonary disease (%)	592 (15.9)
Diabetes mellitus (%)	658 (17.7)
Mean serum albumin (g/L) (SD)	36 (5)
Median Maximum CRP Reading (mg/L) (IQR)	89 (151)
Median time to half maximum CRP (weeks) (IQR)**	0.5 (0.6)
Median Slope CRP to half maximum (mg/L per week) (IQR)**	100 (202)
Median Time to CRP less than 50mg/L (weeks) (IQR)***	0.5 (0.6)
Median Slope CRP to less than 50mg/L (mg/L per week) (IQR)***	106 (139)

*n = 2416

**n = 2496

***n = 1858

CRP: C-reactive protein.

**Fig 1:**
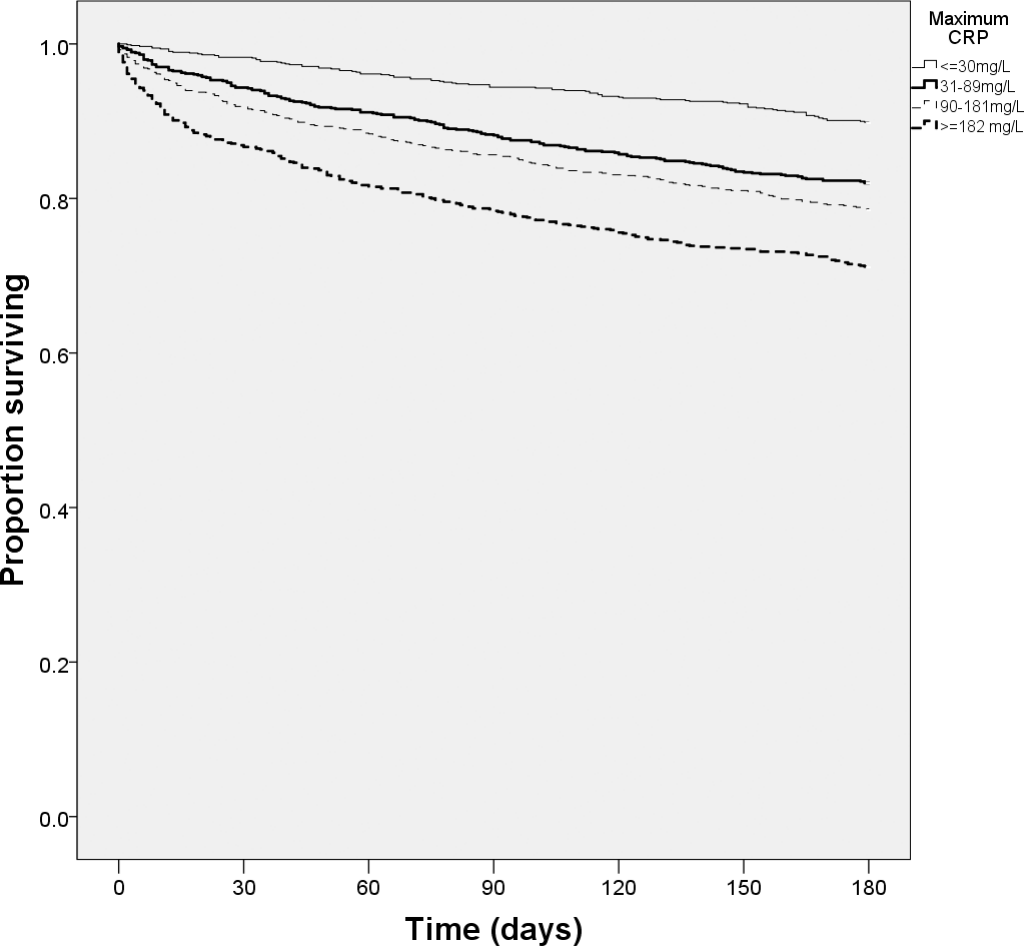
6-month survival by quartiles of maximum C-reactive protein.

**Fig 2:**
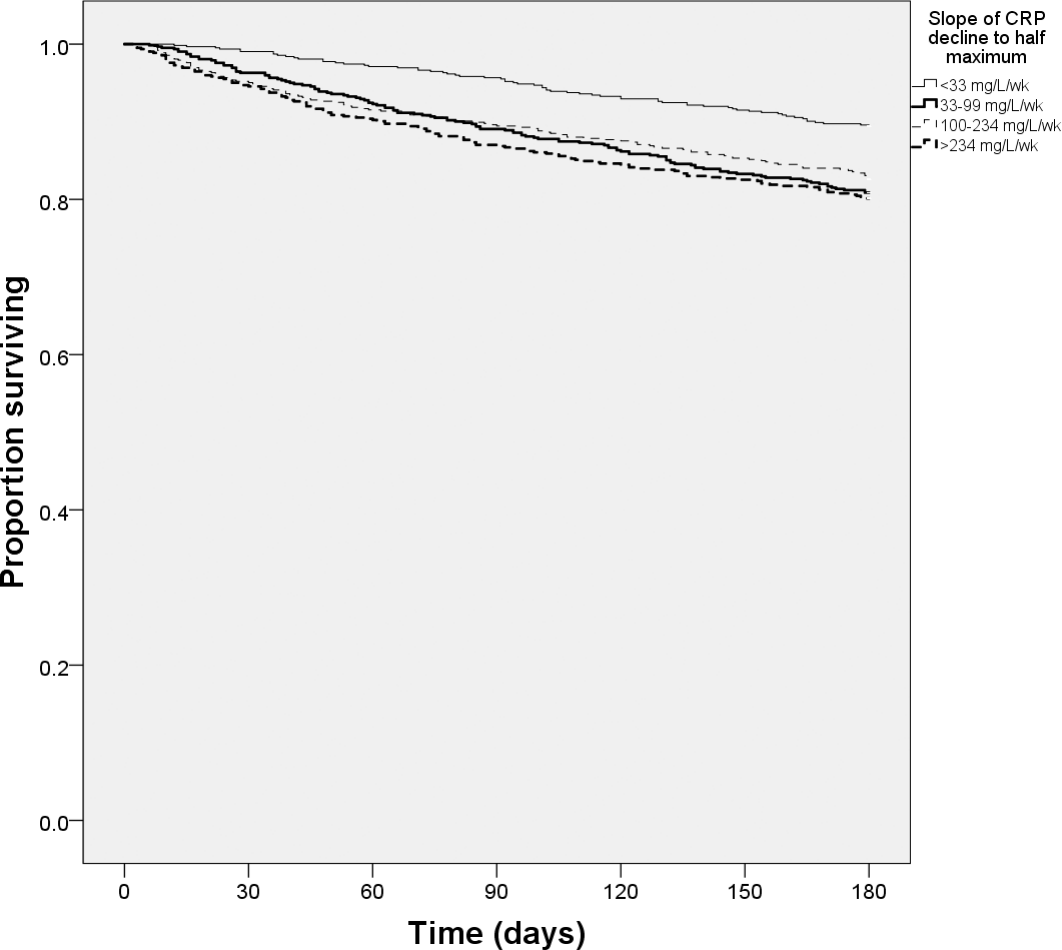
6-month survival by quartiles of slope of C-reactive protein falling to half maximum level.

**Fig 3:**
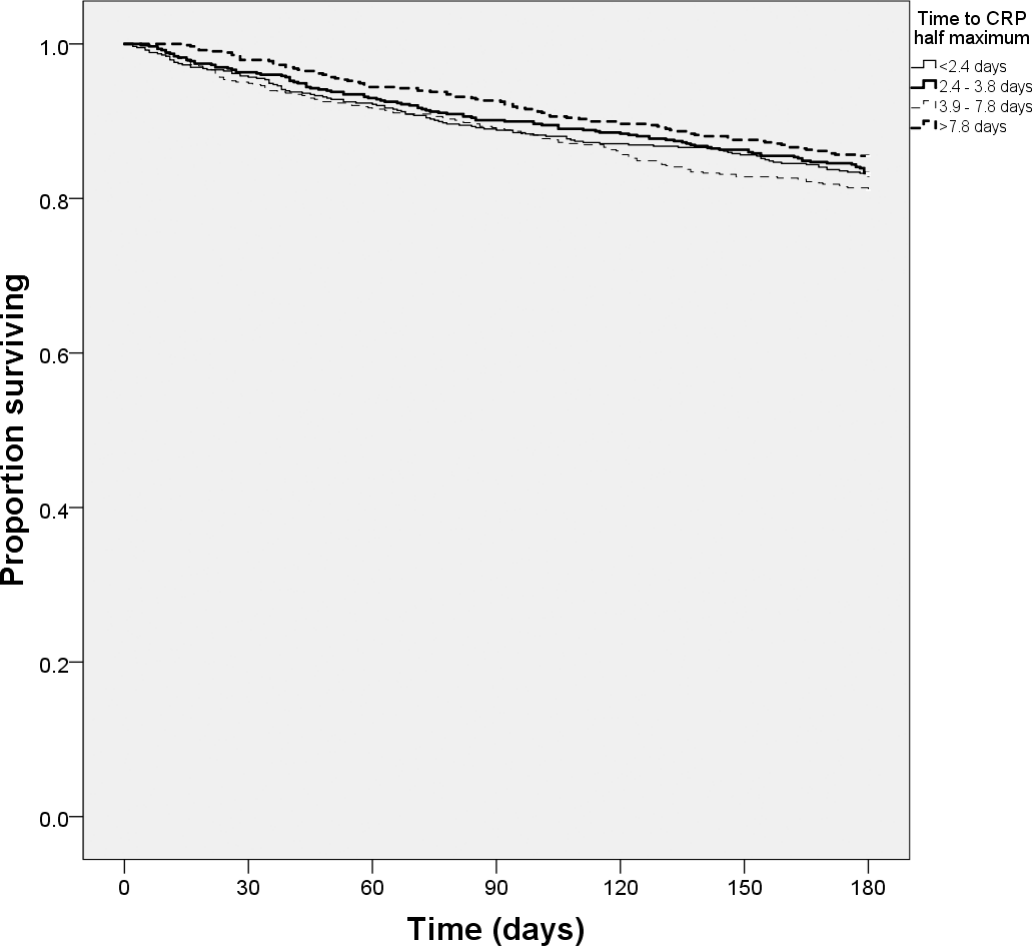
6-month survival by quartiles of time for C-reactive protein to fall to half maximum level.

Unadjusted Kaplan-Meier plots showed that both lower maximum CRP (Fig 1) and reduced slope of CRP fall to half maximum (Fig 2) were associated with higher six month survival (both p<0.001 by log-rank test); longer time taken for CRP to fall was also significant on univariate testing (Table 2)–however, this association was not evident on Kaplan Meier plots (Fig 3). Multivariate Cox regression models (Table 3) showed that the time for CRP to fall to half of the maximum value was the best dynamic CRP index of survival; this remained significant even after adjustment for maximum CRP level and other covariates.

**Table 2:** Univariate Cox Regression Model: Predictors of death within 6 months.

	Hazard ratio (95% CI)	P
Age (per year)	0.98 (0.97, 0.99)	<0.001
Female sex	0.74 (0.64, 0.85)	<0.001
Number of medications	0.78 (0.75, 0.80)	<0.001
Serum calcium (per mmol/L)	2.16 (1.31, 3.57)	0.003
Estimated GFR (per ml/min/1.73m^2^)	1.00 (1.00, 1.00)	0.20
Haemoglobin level (per g/dL)	0.92 (0.89, 0.96)	<0.001
Previous myocardial infarction	1.12 (0.94, 1.34)	0.21
Previous stroke	1.09 (0.84, 1.42)	0.54
Chronic Heart Failure	1.70 (1.39, 2.09)	<0.001
Chronic obstructive pulmonary disease	1.38 (1.15,1.65)	0.001
Diabetes mellitus	0.79 (0.64, 0.97)	0.02
Serum Albumin (per g/L)	0.91 (0.89, 0.92)	<0.001
**CRP measurements**		
Maximum CRP Reading (per mg/L)	1.033 (1.027, 1.039)	<0.001
Time to half maximum CRP (per week)	0.896 (0.836, 0.959)	0.002
Slope of CRP to less than half maximum (10mg/L per wk)	1.008 (1.003, 1.012)	0.001
Time to CRP less than 50mg/L (per week)	0.947 (0.895, 1.002)	0.06
Slope of CRP to less than 50mg/L (10mg/L per wk)	1.003 (0.993, 1.013)	0.52

CRP: C-reactive protein. GFR: Glomerular filtration rate.

**Table 3:** Multivariate Cox Regression Model: CRP measurements as predictors of death within 6 months.

	Unadjusted for covariates		Adjusted for covariates, excluding albumin		Adjusted for covariates, including albumin	
	HR (95% CI)	P	HR (95% CI)	P	HR (95% CI)	P
Max CRP (per 10mg/L)*	1.019 (1.011, 1.028)	<0.001	1.017 (1.008, 1.026)	<0.001	1.013 (1.003, 1.022)	0.008
Time to CRP halfmax (per wk)*	0.915 (0.858, 0.975)	0.006	0.910 (0.851, 0.973)	0.006	0.912 (0.853, 0.976)	0.008
Max CRP (per 10mg/L)*	1.024 (1.014, 1.036)	<0.001	1.020 (1.008, 1.033)	0.001	1.013 (1.000, 1.026)	0.04
CRP Halfmax Slope (per 10mg/L /wk)*	0.999 (0.992, 1.005)	0.66	1.000 (0.993, 1.007)	0.96	1.002 (0.995, 1.009)	0.62
Max CRP (per 10mg/L)**	1.003 (0.991, 1.015)	0.68	1.001 (0.989, 1.013)	0.89	0.997 (0.985, 1.010)	0.68
Time to CRP <50mg/L (per wk)**	0.949 (0.897, 1.004)	0.07	0.948 (0.895, 1.004)	0.07	0.949 (0.895, 1.005)	0.08
Max CRP (per 10mg/L)**	1.004 (0.991, 1.017)	0.55	1.001 (0.987, 1.015)	0.88	0.996 (0.982, 1.010)	0.59
Slope to CRP<50 mg/L (per 10mg/L /wk)**	1.002 (0.991, 1.013)	0.74	1.005 (0.994, 1.016)	0.41	1.007 (0.996, 1.018)	0.22

*n = 2496

**n = 1858.

No clinically important correlations were seen between indices of CRP recovery and the rate of improvement in physical function during rehabilitation (Table 4), with the exception of weak correlations between higher maximum CRP and worse function on admission and consequent slightly longer length of stay.

**Table 4:** Correlation between CRP markers, Barthel scores and length of stay.

	Admission Barthel		Improvement in Barthel		Length of stay		Improvement in Barthel per day	
	r	p	r	p	r	p	r	p
Max CRP	-0.13	<0.001	0.00	0.93	0.06	<0.001	-0.02	0.35
Time to half max CRP	0.03	0.25	-0.06	0.02	0.01	0.59	-0.03	0.26
Slope to half max CRP	-0.02	0.42	-0.02	0.38	0.00	0.93	0.01	0.71
Time to CRP<50mg/L	0.02	0.42	-0.04	0.09	0.02	0.29	-0.01	0.61
Slope to CRP<50mg/L	0.05	0.07	0.00	0.94	-0.05	0.03	0.04	0.13

CRP: C-reactive protein.

## Discussion

Our results show that dynamic changes in CRP levels, as well as peak CRP levels, predict mortality in older hospitalised patients at 6 months. Whilst higher CRP correlated with higher mortality as would be expected, the finding that a longer recovery time from peak CRP was associated with lower mortality was a surprising finding. The first association is consistent with more severe illness producing greater inflammation, causing increased damage across multiple organ systems. This would be expected to reduce physiological reserve, increase frailty and therefore lead to increased mortality [13]; findings that are consistent with the relation between high CRP and adverse outcomes seen across multiple organ systems [13–17]. (14)(15)(16) (17).

Our second finding appears counterintuitive at first, but may reflect more complex physiological processes. Longer periods of raised CRP levels would indicate an ongoing inflammatory response, and therefore ongoing damage. However, it might also be the case that a longer response represents a marker of a more robust immune system. Alternatively, prolonged recovery periods may allow for activation of further defence mechanisms (such as antioxidant pathways or immune priming). This concept of allostatic preconditioning is not unprecedented; documented examples include myocardial ischaemia-reperfusion injury and regular exercise [18–19].

The paradigm of regular exercises offers yet another explanation–repeated stressors can induce anti-inflammatory effects. Interestingly, CRP levels also mirror this pattern of ‘downregulation.’ CRP peaks the day after strenuous exercise and lower resting levels are exhibited in individuals who exercise regularly [6, 19]. Not all of the pathways activated by CRP are pro-inflammatory; it also exerts anti-inflammatory properties including prevention of neutrophil superoxide production, reduction of neutrophil endothelial adherence via L-selectin downregulation, and stimulation of mononuclear IL-1 receptor antagonist synthesis. Such mechanisms might be important in ensuring that the acute inflammatory response is fully deactivated after an episode of illness. As an alternative to the above putative causal mechanisms, rates of change of CRP may of course simply be a marker of, rather than an active contributor to, resilience [20].

Indices of CRP recovery slope showed that a more rapid fall in CRP per unit time was associated with higher six-month mortality. This association was not sustained in multivariable models include maximum CRP however. Because maximum CRP forms part of the calculation of such recovery slopes, these indices were highly correlated with maximum CRP–the higher the maximum CRP, the steeper the recovery slope. Slope of recovery indices were therefore measuring a similar construct to maximum CRP, hence they did not remain as independent predictors in our models.

The overwhelming lack of association seen between CRP indices and measures of physical function, as indicated by baseline and longitudinal changes in Barthel scores for Activities of Daily Living (ADLs), deserves further comment. There was a significant, but weak, negative association between maximum CRP levels and admission Barthel scores and length of hospital stay. This is unsurprising as a higher maximum CRP is likely to signify more severe illness, resulting in a greater degree of impairment on physical function, and requiring a longer recovery period. Given that inflammatory pathways, as well as oxidative stress, are well documented drivers of sarcopenia (the age-related loss of muscle mass and strength), one may have expected a greater degree of association between indices of CRP recovery and rate of improvement in physical function [21]. The lack of association thereof may be explained by the fact that chronic activation of inflammatory pathways and baseline circulating levels of inflammatory markers (such as CRP and IL-6) show such associations, rather than the acute, and appropriate, activation of such pathways in response to an immediate stressor, or to beneficial stimuli such as exercise. Moreover, improvement in Barthel and length of hospital stay are likely to be influenced heavily by treatment strategies and rehabilitation input, making them less accurate markers of intrinsic homeostatic capacity. Similarly, baseline physical function would impact upon both initial Barthel scores, and the scope for improvement. As scores prior to admission were not available, it is difficult to correct for each individual’s pre-existing physical condition and co-morbidities. Furthermore, any ADL score is affected by a large range of physical, psychosocial and cognitive functions, all of which would tend to dilute any association.

### Strengths, Limitations and Future Work

Our study was strengthened by its relatively large sample size. Moreover, the availability of detailed demographic, biochemical and clinical data allowed us to adjust for several important confounding variables. Patients were admitted to rehabilitation after acute medical, surgical or traumatic illness, enhancing the generalisability of our results. Choosing to base CRP indices on rate of recovery, and correcting for maximum CRP levels in analyses, enabled us to model an individual’s intrinsic homeostatic capacity and compensate for variation in maximal CRP levels that may reflect illness severity rather than resilience.

However, there were also numerous limitations to our study. Firstly, most individuals were of white ethnicity. CRP levels vary with ethnicity, making it difficult to extrapolate our findings to other patient populations [16]. There was also a degree of selection bias: individuals in the community who remained healthy, and those who did not survive their hospital admission (arguably the most and least resilient groups respectively) were excluded. CRP did not rise above 50mg/L in half the patients studied, limiting the sample size for some analyses, and the use of routine data means that not all patients had follow up CRP measurements suitable for analysis.

Our findings thus far suggest that a dynamic measure of response to a stressor (halving time of CRP) is an independent predictor of mortality in older people, and may thus be a potential measure of biological resilience. Can these findings be used to assist clinical practice? This may be possible, but further work is necessary to operationalise measures of biological resilience and define whether this construct gives novel prognostic information when compared to existing constructs including frailty, multimorbidity and functional impairment. The work described in this paper represents but the first step along this road.

Our current study focussed solely on six-month mortality as a surrogate end-point for differences in resilience, given the eventuality of death as a final outcome of the spiral of decline often seen in hospitalised older adults. Again, this study was intended as an initial ‘pilot’ investigation, looking at whether or not the concept of resilience was indeed predictive of important clinical outcomes. Given the promising results, future work should explore the associations between resilience and other clinical outcomes, such as changes in specific measures of function, or future hospital admissions. This would allow us to more comprehensively delineate the potential clinical relevance of resilience, and to more accurately compare potential biomarkers to current frailty indices, in order to confirm or refute the idea that resilience and frailty are separate (albeit overlapping) clinical entities. New biomarkers need to be able to provide independent risk prediction regarding outcomes, and provide scope for therapeutic interventions to improve clinical end-points.

Furthermore, a single biomarker is unlikely to accurately reflect the complex physiological processes that constitute homeostasis. To this end, dynamic alterations in other biomarkers (for example sodium trends) should be studied. Sodium levels are acutely sensitive to physiological stressors, and deviation patterns may reflect homeostatic capacity. Other promising biomarkers already routinely measured include albumin, triglyceride, potassium, TSH, uric acid and creatinine levels [22].

Lastly, work is needed to explore the physiological correlates of resilience–not just to understand why slower CRP recovery in our study predicts lower mortality, but to gain better insight into how other cross-system pathologies such as oxidative stress, mitochondrial dysfunction and endothelial dysfunction might combine to produce phenotypes of high and low resilience at a molecular level.
